# Supplementary material for: Wigner solids of domain wall skyrmions
Source: Nat Commun. 2021 Oct 14;12:6006. doi: 10.1038/s41467-021-26306-8 (PMC8516983; doi:10.1038/s41467-021-26306-8)
Supplement: Supplementary file 1 — supplementary information [file 41467_2021_26306_MOESM1_ESM.pdf]

Supplementary Information for

Wigner solids of domain wall skyrmions

Kaifeng Yang, Katsumi Nagase, Yoshiro Hirayama, Tetsuya D. Mishima,

Michael B. Santos & Hongwu Liu*

*email: hwliu@jlu.edu.cn

The PDF file includes:

Supplementary Notes 1-3

Supplementary Figures 1-13

Supplementary Table 1

Supplementary References 1-8

Supplementary Note 1: Edge-channel model for the quantum Hall effect

It is modeled by an edge-channel picture of the quantum Hall effect (Supplementary Fig. 1) to specify sufficient conditions for the appearance of nonlocal resistances[1]. The difference in electrochemical potentials between two terminals is given by $\mu_2 - \mu_1 \approx t_2(1 - t_1)(\mu_\mathrm{B} - \mu_\mathrm{E})/(N - 1)$, where μ_B and μ_E are fictitious chemical potentials for the N-1 edges with perfect transmission through the barrier and the Nth (bulk) channel with transmission probabilities t_1 and t_2, respectively. Apparently, there exists a voltage when the edge and bulk channels are decoupled (i.e., $\mu_\mathrm{B} \neq \mu_\mathrm{E}$) and $t_{1(2)} \neq 1(0)$, no matter whether a net current flows between these two terminals or not and whether they are separated by a short or long distance. With the help of edge states that carry the current to classically inaccessible region, the nonlocal resistance thus occurs even when the voltage probes are separated from the current path over a macroscopic distance.

Supplementary Note 2: Esitmation of bulk current and Hall electric fields in the quantum Hall ferromagnet at filling factor $\nu = 2$

The four-terminal resistance in the bulk-edge model of a quantum Hall conductor with independent edge- and bulk-current components is approximated by $R_{kl,mn} \propto I^N R/I_{kl}$ [where I^N is the sum of the bulk current carried by the highest occupied (Nth) Landau level (LL) in the corresponding segments associated with measurement configurations][2], assuming for simplicity that all segments have the same longitudinal resistance R and the current distribution near a contact is homogeneous. Note that the bulk current I_i^N in segment i of voltage probes characterized by the total current $I_i^t = 0$ is converted from the edge current. Because the edge current flowing into or out of the voltage probe is determined by the direction of B rather than by the polarity of the applied current, the difference between ingoing and outgoing edge currents on opposite sides of the voltage probe leads to a direct current (DC) I_i^N for a given direction of B. Therefore, a DC rather than an alternating current (AC) signal is obtained from the AC nonlocal measurement. The Hall electric field in the bulk state of the Nth LL of segment i is $\mathcal{E}_H = \rho_{xy}^N I_i^N / W$ (where ρ_{xy}^N is the Hall resistivity of the Nth LL and W is the segment width). I^N for the spike of $R_{\mathrm{SD},12}$ (Fig. 1c, main text) has a maximum value of 15.8 nA based on the assumption that the applied current I_SD =

31.6 nA is carried equally by the edge and bulk states. The value of this current corresponds to $\mathcal{E}_H$ ~ 14 Vm^{-1} calculated by W = 30 μm and by $\rho_{xy}^N = h/e^2$ (where h is Planck's constant) that is taken to be independent of the filling factor of the *N*th LL for simplicity. Comparison of $R_{23,14}$ and $R_{SD,12}$ around 13 T in Fig. 1c gives an estimate of an upper bound of I^N~ 0.1 nA and $\mathcal{E}_H$ ~ 0.1 Vm^{-1} for the nonlocal counterpart of the spike according to $R_{kl,mn} \propto I^N R/I_{kl}$. Similarly, the current-independent $R_{23,14}$ around 13 T (Fig. 1f, main text) yields I^N~ 10 nA and $\mathcal{E}_H$ ~ 10 Vm^{-1} for I_{23} = 3.16 μA. From the above discussion it is clear that a small value of I^N/I_{kl} results in relatively low nonlocal resistance that responds more sensitively to subtle change in the sample. This makes it possible to measure the effect of dynamic nuclear polarization (DNP) on the nonlocal resistance at low current accompanied by a small Hall electric field in the bulk, thus allowing for the NRDNMR measurement of the DW structure under equilibrium conditions that is not available by conventional methods.

Supplementary Note 3: Calculation of the effective magnetic field *b**

The effective field b^*measures the energy separation between the two approaching LLs with opposite spins (Fig. 1b, main text), including both single-particle LL splitting and interaction contributions[3,4]. The b^* oriented along the *z* pseudospin direction near ν = 2 is given by $b^* = b_z - U_{z,z}$. Here $b_z = -(E_z - E_c + I_0)/2$ (where E_z and E_c are the Zeeman and cyclotron energies, respectively) with the exchange interaction term $I_0 = {}^1\!/_2 \sqrt{{}^{\pi}\!/_2}\,(e^2/4\pi\varepsilon_0\varepsilon l_B)$ (where e is the electron charge, ε_0 the vacuum permittivity, ε the dielectric constant and l_B the magnetic length) and $U_{z,z} = -{}^1\!/_8 \int_0^\infty dq e^{-\frac{q^2}{2}} \left[L_{n(\downarrow)}\left(\frac{q^2}{2}\right) - L_{m(\uparrow)}\left(\frac{q^2}{2}\right)\right]^2 (1 + e^{-dq})$ (where $L_{n,m}(x)$ is the Laguerre polynomial with $n = 0$ and $m = 1$ for ν = 2 and d is the well thickness) is called the magnetic anisotropic term that depends on the nature of the intersected LLs.

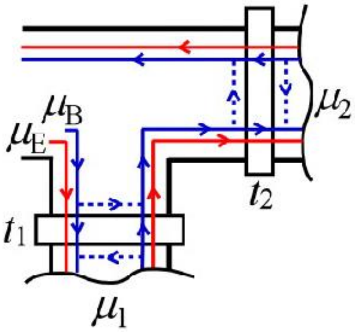

Supplementary Figure 1. **Edge-channel schematics for the quantum Hall effect**. Model of multi-terminal conductors with each rectangular segment representing a barrier that perfectly transmits the *N*-1 edges (red line) but probably backscatters the *N*th channel (blue line) as shown by dashed lines, where *N* is the number of states at the Fermi level. The arrow indicates the direction of electron flow.

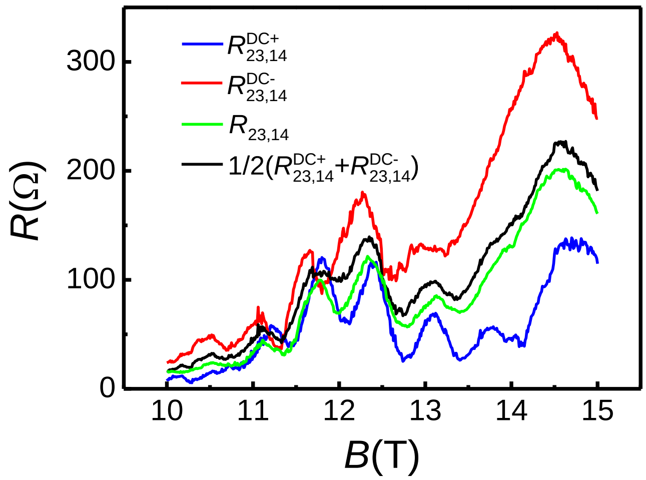

Supplementary Figure 2. **Nonlocal resistance of the quantum Hall ferromagnet at filling factor ν = 2.** $R^{DC+}_{23,14}$ ($R^{DC-}_{23,14}$) for a positive (negative) direct current (DC) and $R_{23,14}$ for an alternating current (AC) as a function of the magnetic field B were measured in a JANIS cryostat at T = 1.6 K and θ = 64°.

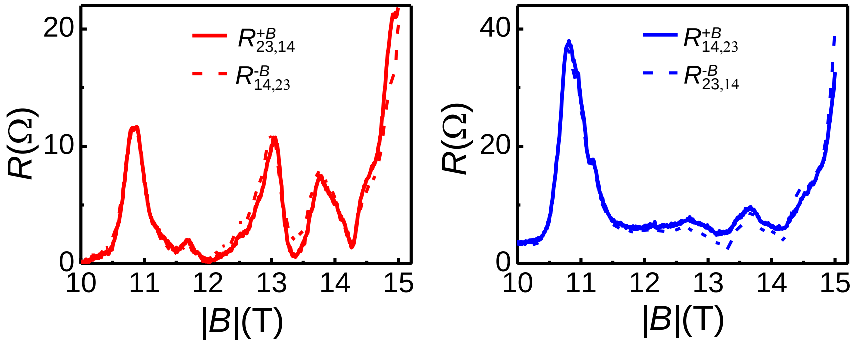

Supplementary Figure 3. **Nonlocal resistance under magnetic-field reversal for small currents.** $R^{\pm B}_{23,14}$ and $R^{\pm B}_{14,23}$ as a function of the absolute value of **B** ($|B|$) at $I_{14(23)}$ = 31.6 nA, T = 1 K, and θ = 64°.

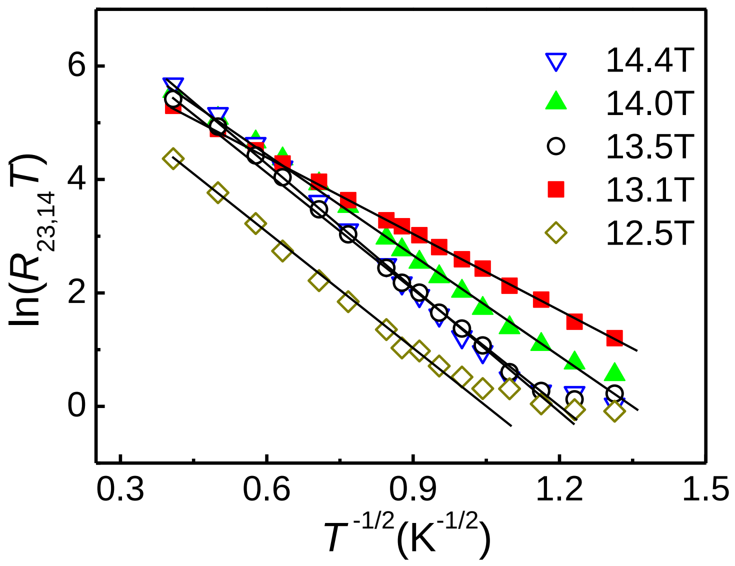

Supplementary Figure 4. **Temperature dependence of the nonlocal resistance of the ν = 2 quantum Hall ferromagnet (QHF) at different magnetic fields.** Data (symbols) are obtained from those in Fig. 1d (see main text). The solid line is calculated by $R_{23,14} \propto \frac{1}{T} exp\left[-\sqrt{\frac{T_0}{T}}\right]$, from which the localization length $\xi = C\frac{e^2}{4\pi\varepsilon\varepsilon_0 k_B T_0}$ (where k_B is the Boltzmann constant, e the electron charge, ε_0 the vacuum permittivity, dielectric constant $\varepsilon = 16.8$, and $C \approx 6$[5]) is derived (Fig. 1e, main text). Note that the variable-range-hopping conductivity $\sigma_{xx}(T) \propto \frac{1}{T} exp\left(-\sqrt{\frac{T_0}{T}}\right)$ is related to the longitudinal (Hall) resistivity ρ_{xx}^N (ρ_{xy}^N) of the Nth Landau level by $\sigma_{xx}^N = \rho_{xx}^N / \left[(\rho_{xx}^N)^2 + (\rho_{xy}^N)^2\right]$ with $\rho_{xy}^N \approx 26\ k\Omega$. Because $R_{23,14} \propto I^N R / I_{23}$ and R is proportional to ${\rho_{xx}^N}^2$, $R_{23,14} \propto \rho_{xx}^N \propto \sigma_{xx}^N$ for $\rho_{xy}^N \gg \rho_{xx}^N$ in our case. That is, the bulk conduction of the ν = 2 QHF changes with temperature in the same manner as the resistance.

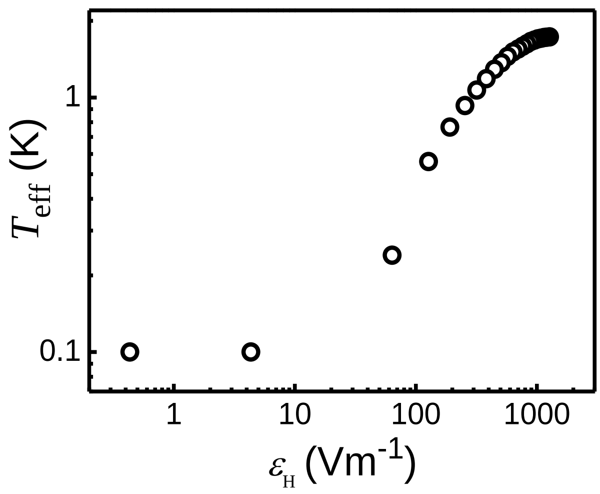

Supplementary Figure 5. **Hall-field-dependent variable range hopping transport.** Effective temperature T_{eff} versus the Hall electric field $\mathcal{E}_H$ for $R_{23,14}$ in Fig. 1d (see main text) at B = 13.5 T and T = 100 mK. T_{eff} is obtained by comparing the measured $R_{\mathrm{SD},12}(T) \equiv R_{\mathrm{SD},12}(I_{\mathrm{SD}})$ point by point, which is related to the existence of a quasi-Fermi level at large $\mathcal{E}_H$[6].

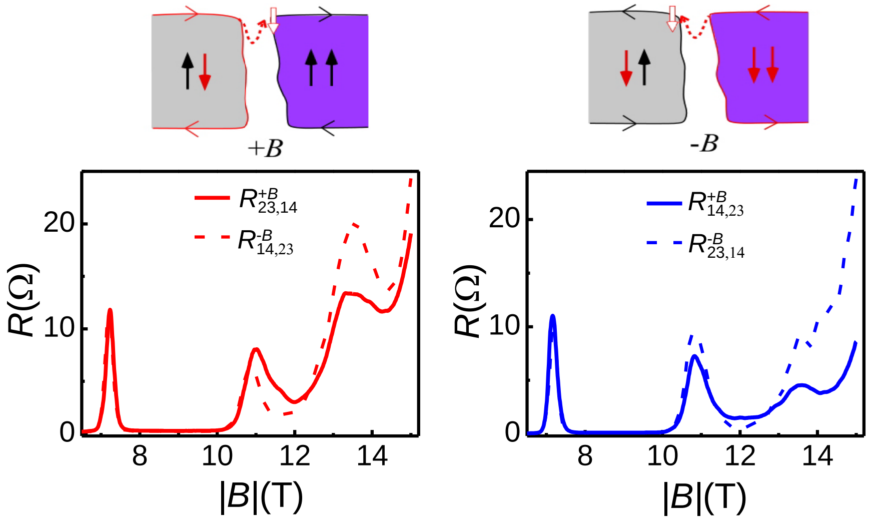

Supplementary Figure 6. **Nonlocal resistance under magnetic-field reversal for large currents.** $R^{\pm B}_{23,14}$ and $R^{\pm B}_{14,23}$ as a function of $|B|$ at $I_{14(23)}$ = 3.16 μA, T = 1 K and θ = 64°. The panel depicts domain structures of the quantum Hall ferromagnet (QHF) formed at filling factor ν = 2. The black (red) solid arrow denotes the spin-up (spin-down) electron. The gray and purple areas denote the spin-unpolarized and spin-polarized domains, respectively, and a domain (DW) occurs in between. A line surrounding each domain represents the edge state that becomes part of an array of domains. The nuclei (hollow arrow) polarized by the electron-spin flip (red dashed arrow) locate on either side of the DW, depending on the direction of edge current flow determined by the sign of the magnetic field B. The polarized nuclei will change static magnetic fields locally via the Overhauser effect, breaking the Onsager relation and thus the reciprocity theorem $R^{+B}_{\mathrm{kl,mn}} = R^{-B}_{\mathrm{mn,kl}}$ in the ν = 2 ($|B|$ ~ 13 T) QHF region. In contrast, the nonlocal resistance of the Landau-level peak outside the QHF region ($|B|$ ~ 7.2 T) is found to obey the reciprocity theorem.

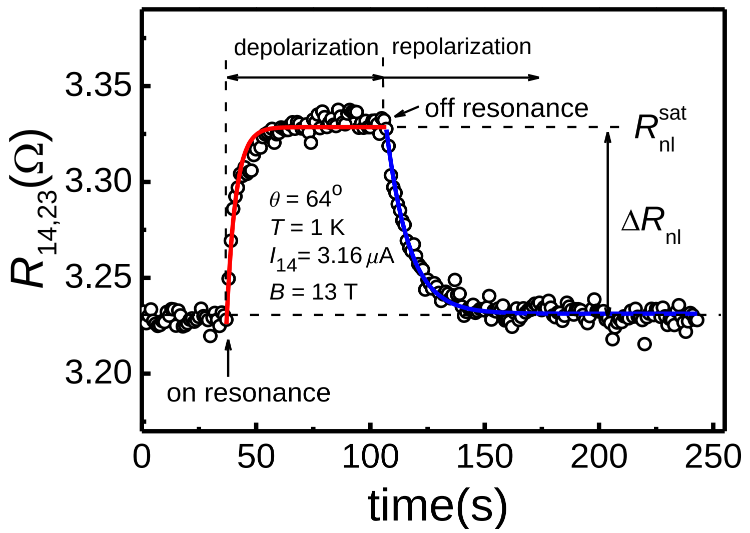

Supplementary Figure 7. **Time dependence of $R_{14,23}$ in the NRDNMR measurement.** The depolarization (repolarization) of nuclei under the condition of on (off) resonance with a continuous-wave radio-frequency matching (mismatching) the resonance frequency of ^{115}In is indicated by an exponential increase (decrease) of $R_{14,23}$. $R_{14,23}$ becomes saturated (R_{nl}^{sat}) at the end of depolarization, resulting in a resistance change ΔR_{nl}. The red (blue) line is an exponential fit to the data in the depolarization (repolarization) process, from which the nuclear spin-lattice relaxation time T_1 and the nuclear spin dephasing time T_2 are derived.

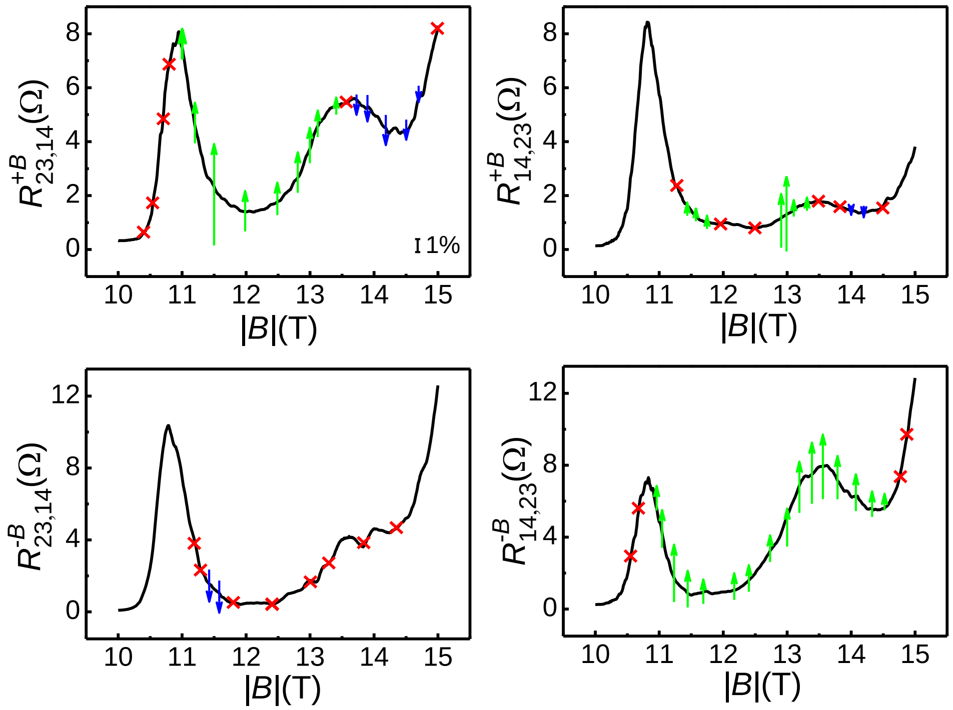

Supplementary Figure 8. **NRDNMR signals of the quantum Hall ferromagnet at filling factor $\nu = 2$.** $R^{\pm B}_{23,14}$ and $R^{\pm B}_{14,23}$ as a function of the absolute value of B ($|B|$) at $I_{14(23)}$ = 3.16 μA, T = 1 K, and θ = 64°. The amplitude and sign of $\Delta R_{\mathrm{nl}}/R^{\mathrm{sat}}_{\mathrm{nl}}$ (Supplementary Fig. 7) are indicated by arrow length (scale bar, 1%) and direction, respectively. Note that the sign, as expected, depends on the relative shift of the nonlocal counterpart of the spike before and after the dynamic nuclear polarization. A cross (×) indicates the absence of NRDNMR signals.

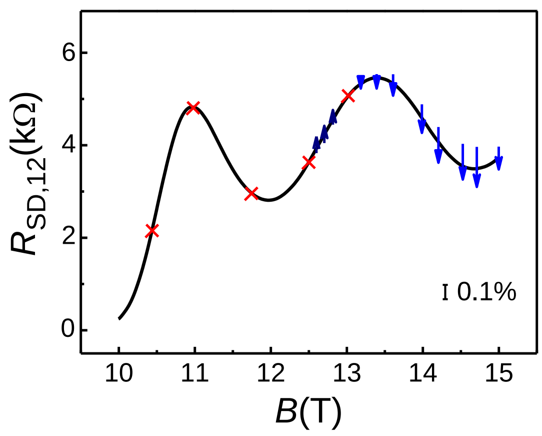

Supplementary Figure 9. **RDNMR signals of the quantum Hall ferromagnet at filling factor ν = 2.** $R_{SD,12}$ as a function of B at I_{12} = 1 μA corresponding to I^N~ 500 nA (see Supplementary Note 2), T = 50 mK, and θ = 64°. The amplitude and sign of $\Delta R_{SD,12}/R_{SD,12}^{sat}$ induced by the dynamic nuclear polarization (DNP) are indicated by arrow length (scale bar, 0.1%) and direction, respectively. Note that the sign is expected to depend on the relative shift of the ν = 2 (B ~ 13 T) spike before and after the DNP. A cross (×) indicates the absence of RDNMR signals.

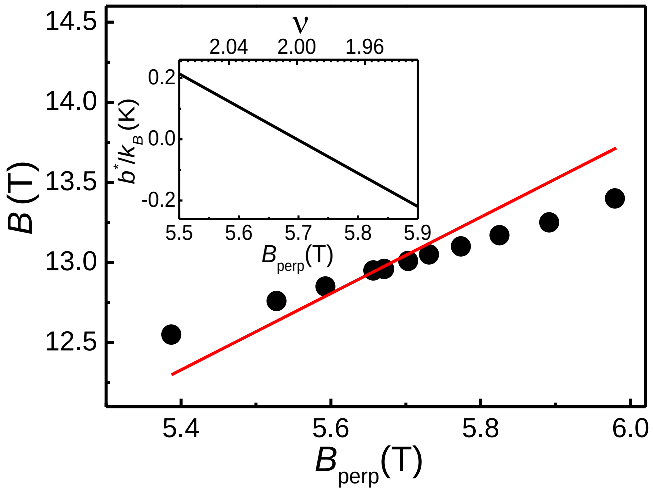

Supplementary Figure 10. **Zero effective field *b** in the (*B*, *B*perp) plane.** Data (dots) in Fig. 2a (see main text) plotted as a function of the magnetic field B and its perpendicular component B_{perp}. The solid line is a theoretical fit (see Supplementary Note 3) showing $b^* = 0$ based on Hartree-Fock calculation of pseudospin anisotropy energy with finite-width corrections[4] using sample parameters[7] of effective mass $m^* = 0.0155$, Landé g factor $g = 54$, dielectric constant $\varepsilon = 16.8$, and well width $d = 20$ nm. This fit does not consider the screening of Coulomb interactions and complex disorder effects, which may be responsible for the deviation of the fit from the data. Inset: b^*/k_B (where k_B is the Boltzmann constant) as a function of B_{perp} (or filling factor ν) at the tilt angle $\theta = 64.12°$.

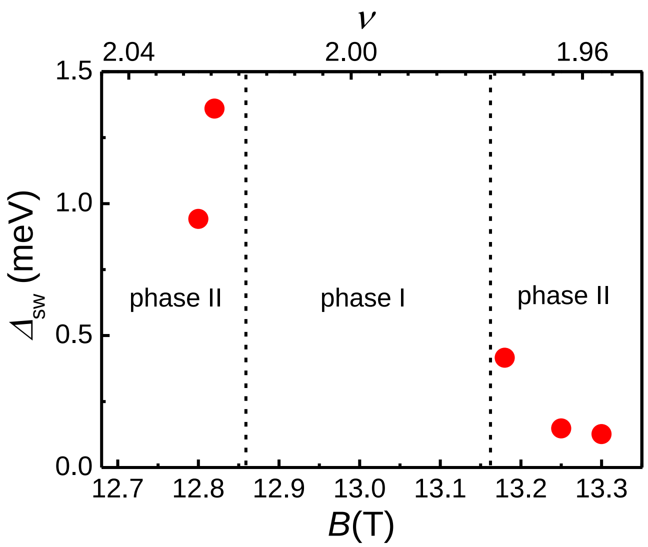

Supplementary Figure 11. **Activation energy Δ_{sw} for the spin-wave (SW)-mediated nuclear relaxation in neutral domain walls (DWs).** Δ_{sw} as a function of the magnetic field *B* (or filling factor ν) in phase II (dots) obtained by a fit to the data of $1/T_1$ versus *T* at different *B* (Fig. 2a, main text) using an Arrhenius law.

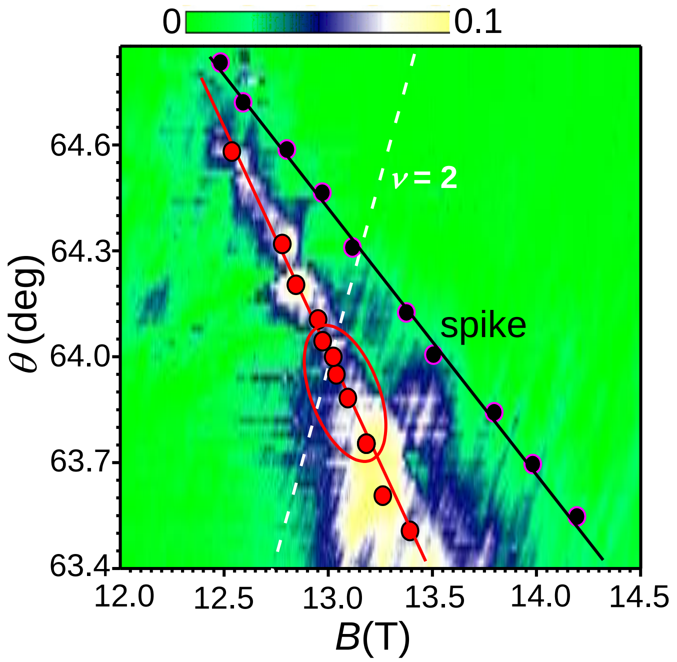

Supplementary Figure 12. **Comparison of the position of maximum $\Delta R_{\mathrm{hys}}/R^{+B}_{14,23}$ and maximum $R^{+B}_{14,23}$ of the quantum Hall ferromagnet at filling factor $\nu = 2$.** Contour plot of the magnetic field B and the tilt angle θ as a function of $\Delta R_{\mathrm{hys}}/R^{+B}_{14,23}$ is the same as that in Fig. 3a (see main text). Dots indicate the position of maximum $\Delta R_{\mathrm{hys}}/R^{+B}_{14,23}$ (red) and maximum $R^{+B}_{14,23}$ (black), respectively. The solid line is a guide to the eye and the dashed line is calculated for the B and θ dependence of $\nu = 2$.

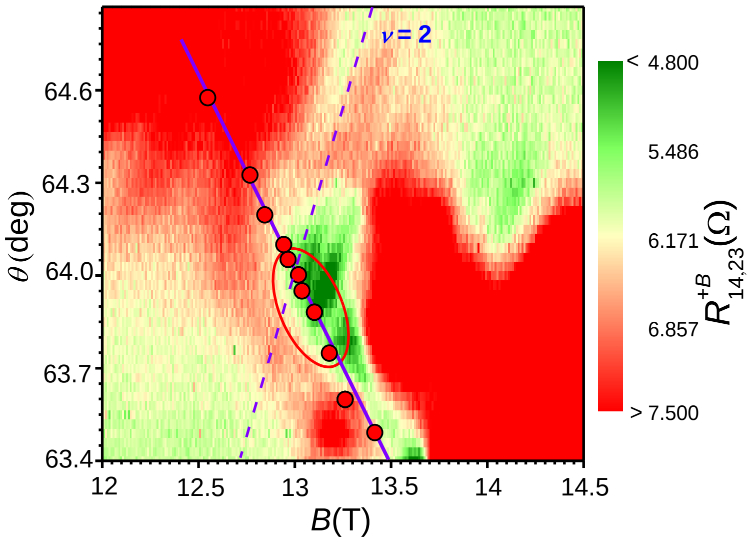

Supplementary Figure 13. **Nonlocal resistance of the quantum Hall ferromagnet at filling factor ν = 2 for small currents.** Contour plot of the magnetic field B and the tilt angle θ as a function of $R^{+B}_{14,23}$ at a field-sweep rate of 1.7 mT/s, I_{14} = 31.6 nA, and T = 1 K. Red dots and the oval correspond to those shown in Fig. 2a (see main text). The solid line is a guide to the eye, and the dashed line is calculated for the B and θ dependence of ν = 2.

Supplementary Table 1. Comparison of Zeeman energy and perturbative ratio α in GaAs 2DEGs and InSb 2DEGs (well width $d = 20\,nm$, B = 13 T, magnetic length $l_B = 256\,Å/\sqrt{B}$). Parameters are taken from Ref. 8 and from our experimental data.

	GaAs	InSb
effective g factor	-0.44	-54
Zeeman energy E_z(meV)	0.3	40
effective mass m^*(in units of m_0)	0.067	0.015
cyclotron energy (meV) $\hbar\omega_c = \hbar\frac{eB}{m^*}$	22.4	100
SOC coefficient γ (eV · $Å^3$)	27	500
SOC energy (meV) $\epsilon_{so} = \gamma\left(\frac{\pi}{d}\right)^2/l_B$	0.1	1.7
perturbative ratio $\alpha = \epsilon_{so}/\hbar\omega_c$	0.0045	0.017

Supplementary References

1. McEuen, P. L., Szafer, A., Richter, C. A., Alphenaar, B. W., Jain, J. K., Stone, A. D., Wheeler, R. G. & Sacks, R. N. New resistivity for high-mobility quantum Hall conductors. *Phys. Rev. Lett.* **64**, 2062-2065 (1990).

2. van Son, P. C., de Vries, F. W. & Klapwijk, T. M. Nonequilibrium distribution of edge and bulk current in a quantum Hall conductor. *Phys. Rev. B* **43**, 6764-6767 (1991).

3. Jungwirth, T., Shulka, S. P., Shayegen, M & MacDonald, A. H. Magnetic anisotropy in quantum Hall ferromagnets. *Phys. Rev. Lett.* **81**, 2328-2331 (1998).

4. Jungwirth, T. & MacDonald, A. H. Pseudospin anisotropy classification of quantum Hall ferromagnets. *Phys. Rev. B* **63**, 035305 (2000).

5. Polyakov, D. G. & Shklovskii, B. I. Conductivity-peak broadening in the quantum Hall regime. *Phys. Rev. B* **15**, 11167-11174 (1993).

6. Polyakov, D. G. & Shklovskii, B. I. Variable range hopping as the mechanism of the conductivity peak broadening in the quantum Hall regime. *Phys. Rev. Lett.* **70**, 3796-3799 (1993).

7. Yang, K. F., Liu, H. W., Mishima, T. D., Santos, M. B., Nagase, K. & Hirayama, Y. Nonlinear magnetic field dependence of spin polarization in high-density two-dimensional electron systems. *New J. Phys.* **13**, 083010 (2011).

8. Winkler, R. *Spin-Orbit Coupling Effects in Two-Dimensional Electron and Hole Systems* (Springer, Berlin, 2003).
